# Gold.—Number V.

**Published:** 1859-07

**Authors:** 


### GOLD.—Number V.

BY PROF. AUSTIN.

Before speaking of the various uses of Gold in its pure and in its alloyed condition, we propose to devote some space to the peculiar method of valuation, sanctioned by long usage among workers in this precious metal; but which will, most parbably, in time, yield to the greater convenience of the decimal system, now adopted in the mints of France and America. As yet, however, the fineness of American gold is much more familiarly known to dentists by its valuation in carats than in decimals, notwithstanding the superiority of the latter method.

The term *carat*, or *karat*, originally designated an Abyssinion bean. Being very uniform in size, and undergoing scarcely any loss by drying, they came to be used as the standard of weight, in Africa for gold and in India for diamonds. Each carat was divided into 4 grains, of which 74 are nearly equal to 72 grains troy. This system of carats and grains is still used in the valuation of diamonds. But in the case of gold, the term *carat* implies, not so much any actual weight, as a fractional division, of which 24 go to make a unit. *Twenty-four carats fine* expresses the unity of pure gold, and signifies, not the specific weight of any given mass, but only that, in the 24 imaginary parts into which it may be supposed to be divided, there is no alloy.

The gold assayer takes as his unit or *integer* 6 or 12 grains troy. This small quantity is most convenient for purposes of assay, and these particular numbers are used for convenience of calculation. This 6 or 12 grains is called by the English assayer an *assay pound* and is by him divided into 24 carats and each carat again into quarters and sixteenths. The assayer of silver takes 18 to 36 grains troy for his assay pound and divides it into 12 ounces, each ounce into 20 pennyweights and these again into half-pennyweights—making, for the silver assay pound 480 divisions or *reports* and for the gold assay pound 384 *reports*. On the Continent of Europe the division of the assay pound for gold is different from the English.

In the English mint the term carat expresses no given weight, but merely degrees of fineness of which 24 indicate, purity. The carat is subdivided into quarters and these again into eighths—making to each carat 32 parts, 768 of which represent pure gold.

These varying, complicated and arbitrary systems are the relict of an age which delighted in intricate and perplexing mysteries. They are gradually yielding before the scientific demand for uniform and universal formulæ. Instead of each trade having its own peculiar weights and measures, there must come to be one standard for all business and ultimately one for all the leading nations of the earth. Instead of one measure for cloth, another for length and a third for land; one measure for wine another for beer and another for grain; one weight for the apothecary and another for the grocer: one standard for France a second for England and a third for America, there will be one uniform standard for all, based upon the Decimal system—England has adopted it in her silver assay, America in her currency, France in her system of weights and measures, and partially in her currency: and there are now movements in agitation for a uniform basis upon which these three great nations may unite. But until this desirable end is consummated we shall continue to talk of gold in the old-fashioned way, and even while our mint reports the fineness of its coin at 900-thousandths, we find it more usual to reckon it at 21 6 carats.

For the dentist it is convenient and quite admissible to take the terms *carat* and *grain* as equivalent, since the one bears the same relation to the pennyweight that the other does to purity. Thus the pennyweight of gold that contains 24 grains of pure gold is 24 carats fine: and 18 carat gold has in each pennyweight 18 grains pure gold and 6 of alloy. We thus get rid of the arbitrary values and subdivisions of the old "carat" system, substituting the familiar value of the Troy system. The still more convenient decimal system represents the unit of purity by 1000. American coin contains 1-10th alloy and is represented by 908 under the decimal system, its carat value is 21.6. English coin contains 1-12*th* alloy; 916.6 is its decimal, and 22. its carat value. The following short table will give a comparative view of these three ways of estimating the proportion of gold in a given alloy—column *first* represents the fractional proportion of gold, column *second* the carat value, column *third* the decimal equivalent:

TABLE.

| *Fraction.* | *Carat.* | *Decimal.* | *Fraction.* | *Carat.* | *Decimal.* |
|---|---|---|---|---|---|
| 1-8th | 3. | 125. | 5-8th | 15. | 625. |
| 1-6th | 4. | 166.6 | 2-3rd | 16. | 666.6 |
| 1-5th | 4.8 | 200. | 3-4th | 18. | 750. |
| 1-4th | 6. | 250. | 4-5th | 19.2 | 800. |
| 1-3rd | 8. | 333.3 | 5-6th | 20. | 833.3 |
| 3-8th | 9. | 375. | 7-8th | 21. | 875. |
| 2-5th | 9.6 | 400. | 9-10th | 21.6 | 900. |
| 1-2lf | 12. | 500. | 11-12th | 22. | 916.6 |
| 3-5th | 14.4 | 600. | | | |

Applying this table to the choice of gold plate for dental purposes, we may select anywhere between 18 carats, containing $\frac{3}{4}$ pure gold, or 750-1000ths and 21.6 carats, containing 9-10ths pure gold or 900-1000ths. Of the relative merits of different finenesses and the most suitable metal for alloy we shall speak subsequently.

I conclusion of the subject of carat valuation it remains to give some rules for finding—1st, how much alloy to add in order to reduce the gold to a given standard; 2dly, what will be its fineness after the addition of a given amount of alloy. All correct rules may be deduced from the following formula which we have given without entering into any explanation of the original equation from which they are derived. Representing the amount of alloy by A—the weight to be alloyed by G—the higher carat by C—the lower carat by c—then

$$(1)\ A = G \div \frac{c}{C - c}$$

that is, to reduce a given weight of gold from one carat to a lower:—*Subtract the lower carrat from the higher; divide the lower carat by the difference; with this quotient divide the weight of the gold and it will give the amount of alloy.*

$$(2)\ A = \frac{CG}{c} - G$$

that is—*multiply the weight of gold by its carat: divide by the carat to which it is to be reduced; subtract the weight of the gold and the remainder will be the weight of alloy required.*

Either rule is equally accurate, being both derived from the same original equation, and both are perfectly correct. The latter is an old rule; the former was first given by the writer, some years since, in the New York Recorder. It can be worked out more quickly than the frst rule, in most cases. The following problems useful to the dentist are given in illustration of these rules—Reduce a double eagle (516 grs.—21.6 carats) to 20 carats; also to 18 carats. By the 1st rule 516 is to be divided by [c divided by C — c] which in the first case is 20 ÷ 1.6 = 12.5, in the second case it is 18 ÷ 3.6 = 5; giving 41.28 grains the alloy required for the 20 carat gold and 108.2 grains for the 18 carat. By the 2nd rule 516 × 21.5 = 11145.6 which divided by 20 = 557.28 from which take 5.16 and we have 41.28. Again 516 × 21.6 = 11145.6 which divided by 18 = 619.2 from which take 516 and we have 103.2; the working of the first sum may be shortened a little by multiplying by 24 and dividing by 5—and of the second sum still more by multiplying by 1.2 and dividing by 1; still the first rule will be found almost invariably the shortest. Reduce four sovereigns (490 grs.—22 carats) to 20 carats, also to 19 carats. 20 ÷ 2 = 10 the divisor for the first sum, and gives 49 grains for answer; 19 — 3 = 6.33 the divisor for the second sum, and gives 77.3. In this last sum both rules work equally well.

The gold coins of different countries vary in fineness. The majority are about 21.5 carats. A few are under 21. such as nearly all the South American coins, except the Brazilian moidore and half-Joe.

The Brazilian and Portuguese coins, Russian rouble and the Austrian and Belgian sovereign are all nearly, some exectly, 22 carats. The Italian onzia and sequin, the Russian Austrian and German ducat are over 23 carats and some of them almost perfect pure.

As the coins of the United States and England are those almost universally used by the Dentist we subjoin some more precise statements regarding them.

The Eagle coined prior to July 31, 1834, weighed 270 grains, and was 22 carats fine, containing 274.5 grains of gold (half and quarter eagle in proportion.) Since July, 1844, the standard fineness has been 900 thousandths or 21.6 carats; the eagle, weighing 258 grains (12 grains less) and containing 232 grains pure gold. (Double eagle and the gold dollar in the same proportion.)

The alloy used in the gold coin was formerly 3 parts copper, 1 part silver—that is in 1000 parts of coin, present standard, there was 600 pure gold, 75 copper, 25 silver—these coins are recognized by their yellow color; (the California coin containing *no* copper, is still yellower and very soft,) of late years the alloy used is 99 parts copper to 1 part silver, the latter being used chiefly to soften the color. These coins are distinguished by their reddish color and resemble more nearly the English sovereigns.

The pound sterling is represented by the coin called the *sovereign*, whose legal standard is eleven-twelfths (or 916$\frac{3}{8}$ thousands) fine, and at the rate of 46.78 pieces to a pound troy; equal to 123$\frac{1}{8}$ grains to each piece. The coinage of sovereigns commenced in 1816. Before that time the principal coin was the *guinea*, of one pound and one shilling, (21s) which was on the same legal basis and which ceased to be coined when the exact piece was introduced. Although the term *guinea* is still in familiar use there, the actual coin is seldom seen, and need not be further spoken of except to say that the pieces are so much and so irregularly worn that they can only be taken by weight, their average fineness being 915$\frac{1}{2}$. From 1816 to about 1851 the average fineness of sovereigns was 915$\frac{1}{2}$, with great regularity. Since that date the fineness has been more exactly conformed to the legal standard and is reported by us at 916$\frac{1}{2}$. The average weight of the older pieces 0.251 ounce, and the value \$4,84.8; new pieces 0.256$\frac{3}{4}$, or 123$\frac{1}{6}$ grains; value \$4,86.3. There are also half-sovereign, and some double sovereign have been coined.

